# Case Report of Ectopic Liver on Gallbladder Serosa with a Brief Review of the Literature

**DOI:** 10.1155/2016/7273801

**Published:** 2016-10-10

**Authors:** Vishnu R. Mani, Mohammad S. Farooq, Utsav Soni, Aleksandr Kalabin, Ajai S. Rajabalan, Leaque Ahmed

**Affiliations:** ^1^Department of Surgery, New York University School of Medicine, New York, NY 10016, USA; ^2^Department of Surgery, Columbia University College of Physicians and Surgeons at Harlem Hospital Center, New York, NY 10037, USA; ^3^Department of Internal Medicine, Western Reserve Health Education/NEOMED, Youngstown, OH 44501, USA

## Abstract

This case describes an intraoperative incidental finding and surgical removal of ectopic liver tissue attached to the gallbladder during a standard laparoscopic cholecystectomy for acute cholecystitis. These anomalies are rare, with interesting associations and possible clinically relevant complications. The details of the case, along with a brief literature review of embryology, common ectopic sites, and associations/complications, are presented in this paper. Since laparoscopic cholecystectomy is a very common procedure, it is important to increase vigilance of ectopic liver tissues during surgeries to minimize complications and provide optimal management.

## 1. Introduction

Ectopic liver is a rare finding with a known incidence of 0.24–0.48% and a prevalence rate of 0.47% [1]. It is imperative for clinicians and surgeons to have adequate knowledge of ectopic liver and its inherent complications, associated pathologies, and surgical challenges. In this paper we discuss a case of ectopic liver that was not detected on radiologic imaging but was seen attached to gallbladder wall during laparoscopic cholecystectomy, creating challenges in visualization and dissection during the operation.

## 2. Case Report

A 56-year-old Hispanic male presented to the emergency department with acute onset of epigastric pain associated with meals and had similar episodes in the past with vomiting, 9/10 sharp localized pain to the epigastric region without radiation, and absent associated symptoms. Patient had no significant past medical history except for removal of a cyst from his upper chest. TA vitals were 163/98 mmhg, 97.3 F. The laboratory evaluation of patient sera indicated elevated leukocyte count and altered liver enzymes. Computed tomography scan of abdomen demonstrated distended gallbladder with thickening of the wall and presence of stones; however there was no evidence of pancreatitis and inflammatory changes. Fatty liver was also noted.

Patient was taken for a standard laparoscopic cholecystectomy, prepped, and draped in the universal standard sterile fashion. The abdominal cavity was entered using Hasson's technique, CO_2_ insufflated to 15 mmhg. Four additional ports were created: one port 11 mm to the right of falciform ligament, one 10 mm port in the subxiphoid region, and two 5 mm ports in the right upper quadrant and flank, respectively.

**Figure 1:**
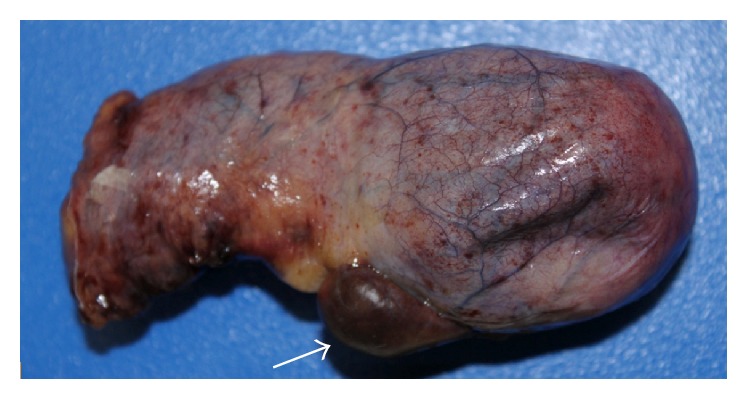
Gross image of resected specimen with ectopic liver tissue present on gallbladder (arrow).

**Figure 2:**
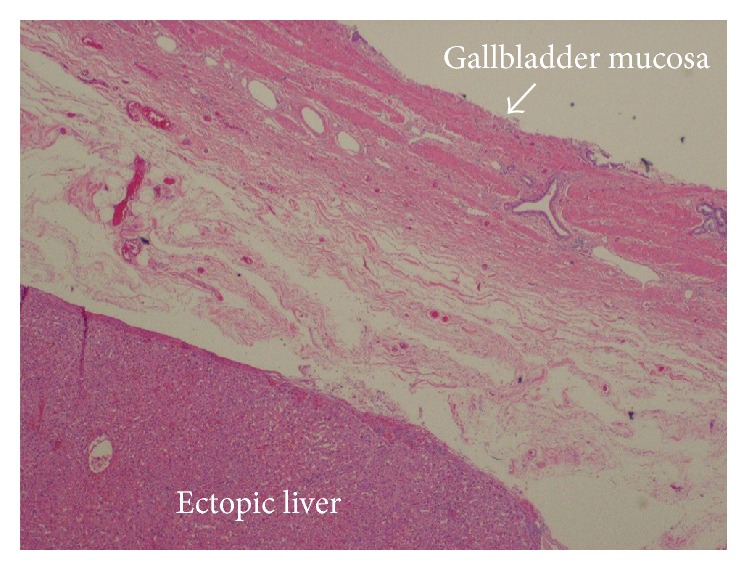
Low power histological image of gallbladder tissue and ectopic liver tissue.

**Figure 3:**
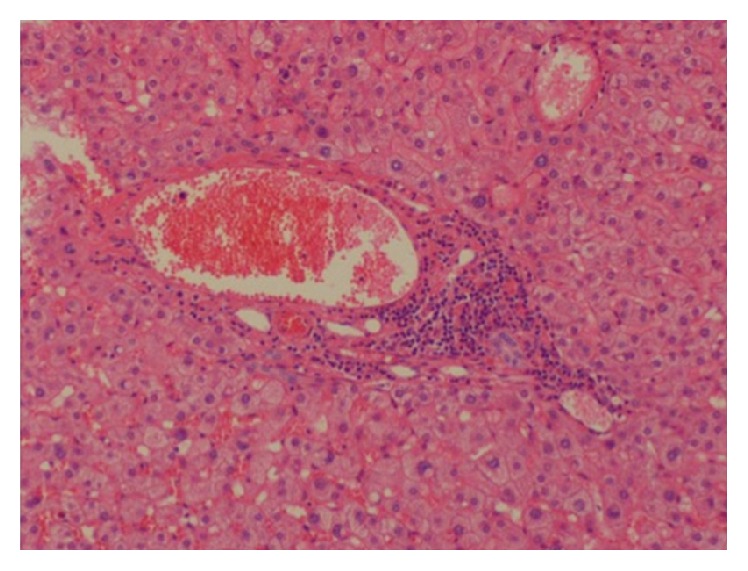
High power histological image of ectopic liver tissue.

The gallbladder was retracted cephalad while Hartmann's pouch was retracted laterally. It was then noted that an ectopic tissue was present on the gallbladder wall (Figure 1). First the gallbladder was mobilized using the standard procedure. Calot's triangle was dissected, the cystic artery was clipped and incised, and the cholangiographic catheter along with fluoroscopy was used to confirm that there was no obstruction in the biliary ducts. Electrocoagulation was then used to remove the gallbladder from the liver. Careful dissection was done to prevent damage to the ectopic tissue with the electrocautery. Once the gallbladder was fully mobilized the pedicle of the lobular ectopic tissue was ligated and transected from the gallbladder fossa on the liver bed. The specimen was removed via endo pouch and sent for histopathological examination. The tissue was confirmed to be ectopic liver (Figures 2 and 3).

## 3. Discussion

Ectopic liver is a rare entity with less than 100 reported cases in the literature [2]. Furthermore in a study of 5500 autopsies, only 13 cases of ectopic liver were found, of which 3 were attached to the gallbladder [3]. Furthermore, in a laparoscopic series of 1060 cases, the prevalence of ectopic liver was found to be 0.47% [4]. Ectopic liver can be found in intra-, extra-, and retroperitoneal sites like the diaphragm, hepatic ligaments, omentum, stomach, retroperitoneum, and thorax, but the gallbladder is the most common site. Ectopic livers are a result of anomalous embryological developments and several theories have been proposed in our review of the literature. One explanation for the development of ectopic liver includes the migration of pars hepatica of the liver bud to distant sites [5]. Another explanation involves trapping of the hepatocyte destined mesenchyme and entrapment of cell nests in various locations, such as the region of the foregut following the diaphragm or umbilical ring closure, or to the GB as accessory liver with atrophy or regression of the connection to the main liver [6, 7]. Furthermore, dorsal budding of hepatic tissue before pleuroperitoneal canal closure has also been proposed as a cause of ectopic liver [8]. Although the ectopic tissue is usually attached to the serosa of the gallbladder, the close relationship between the cystic portions and the parenchymal cell cords of the primitive liver may explain why ectopic liver can occur in the gallbladder wall as well [5, 8]. It can also occur in the gallbladder lumen [9].

Ectopic liver is a rare incidental finding on radiologic imaging, but due to its compressive effect on the gall bladder it can present as a case of acute cholecystitis, as evident in our case [10]. The complications of an ectopic liver include torsion, peritoneal bleeding, fatty change with evolution to cirrhosis, and malignant degeneration to hepatocellular carcinoma [1]. Malignancy arising in the ectopic liver tissue should not considered as an occult metastasis from a primary hepatocellular carcinoma [11].

Arakawa et al. report in their review of the literature that there were 21 HCC cases related to ectopic liver. Patients who had ectopic liver tissue on the gall bladder were less susceptible to HCC development as comparable to other localizations outside the gall bladder. This study also revealed that only 1 out of 42 ectopic liver tissues localized on the gallbladder was diagnosed as malignant [12]. The malignant change in the ectopic liver tissue is due to insufficient drainage of bile with or without adequate blood supply to the liver. It is important to note that most of the ectopic liver tissue can present with its independent blood supply. From a surgical standpoint it is important to delineate the blood supply of the ectopic liver as it can cause uncontrollable hemorrhage during resection if it derives its blood supply from the liver parenchyma. In cases of an ectopic liver tissue associated with GB it derives its blood supply in one of the following three ways: vascular pedicle arising with or without its own vein from the liver parenchyma; an artery arising from the cystic artery; and vascular structures embedded in the mesentery lying adjacent to the liver parenchyma [10]. Furthermore, congenital anomalies such as omphalocele, agenesis of the caudate lobe, bile duct cysts, and biliary atresia have been reported as associations with ectopic liver [13].

In conclusion we would like to emphasize the importance of being vigilant of ectopic livers, their complications, and the potential surgical risks as described above, including increased operative time and the need to follow up on such patients to rule out any possible associations. With the limited literature available ectopic livers can be missed on radiological exam, as in our case.

Since cholecystectomy is a routinely performed surgery, surgeon awareness will help combat any potential intraoperative complications. Case reports such as ours are an addition to the knowledge base and would help the surgical community recognize and anticipate anomalies of liver, ultimately providing patients with optimal medical care.
